# Physical Interaction of T Cells with Dendritic Cells Is Not Required for the Immunomodulatory Effects of the Edible Mushroom *Agaricus subrufescens*

**DOI:** 10.3389/fimmu.2016.00519

**Published:** 2016-11-22

**Authors:** Ruud H. P. Wilbers, Lotte B. Westerhof, Jan van de Velde, Geert Smant, Debbie R. van Raaij, Anton S. M. Sonnenberg, Jaap Bakker, Arjen Schots

**Affiliations:** ^1^Plant Sciences Group, Wageningen University and Research Centre, Wageningen, Netherlands

**Keywords:** immunomodulation, mushroom, PAMP, PRR, dendritic cell, T cell

## Abstract

Mushrooms are well known for their immunomodulating capacities. However, little is known about how mushroom-stimulated dendritic cells (DCs) affect T cells. Therefore, we investigated the effect of mushroom compounds derived from seven edible mushroom species on DCs, their fate in DCs, and the effect of the mushroom-stimulated DCs on T cells. Each mushroom species stimulated DCs in a different manner as was revealed from the DC’s cytokine response. Assessing DC maturation revealed that only one mushroom species, *Agaricus subrufescens*, induced complete DC maturation. The other six mushroom species upregulated MHC-II and CD86 expression, but did not significantly affect the expression of CD40 and CD11c. Nevertheless, mushroom compounds of all investigated mushroom species are endocytosed by DCs. Endocytosis is most likely mediated by C-type lectin receptors (CLRs) because CLR binding is Ca^2+^ dependent, and EGTA reduces TNF-α secretion with more than 90%. Laminarin partly inhibited TNF-α secretion indicating that the CLR dectin-1, among other CLRs, is involved in binding mushroom compounds. Stimulated DCs were shown to stimulate T cells; however, physical contact of DCs and T cells is not required. Because CLRs seem to play a prominent role in DC stimulation, mushrooms may function as a carbohydrate containing adjuvant to be used in conjunction with anti-fungal vaccines.

## Introduction

The functioning of the immune system may be impaired as a consequence of dietary deficiencies or imbalances and may thereby negatively impact health. Edible mushrooms are appreciated as health promoting food, notably having antitumor and immunomodulatory effects (1, 2). Mushrooms contain compounds that stimulate the immune system as well as prevent or cure infectious diseases, cancer, allergies, autoimmune, and inflammatory disorders (3–5). Various compounds including polysaccharides, mainly α- and β-glucans, saccharopeptides, (glyco)proteins, and various low molecular weight compounds such as terpenes and phenols, contribute to these medicinal effects (2, 6). Of these compounds the role of β-1,3-glucans with β-1,6 branches is best documented, although the role of other compounds is increasingly appreciated (3, 4, 6, 7).

Immunomodulation by mushrooms mainly takes place through macromolecules that function as pathogen-associated molecular patterns (PAMPs). Pattern recognition receptors (PRRs) on innate immune cells recognize these PAMPs, and these innate cells may subsequently interact with adaptive immune cells. Different classes of PRRs such as toll-like receptors (TLRs), complement receptor 3 (CR3), and C-type lectin receptors (CLRs) play a role in recognizing mushroom PAMPs (2, 8–10). The macromolecular composition of mushrooms is complex, and consequently, different classes of PRRs are involved in binding different PAMPs. TLRs bind glyco- and/or lipoproteins, CR3 binds β-glucan, whereas CLRs bind molecules carrying glyco-moieties such as glycoproteins, mannans, and glucans (10–12). The mixture of compounds likely results in the simultaneous binding of different PRRs, leading to additive and possibly even synergistic effects (13). Dendritic cells (DCs) play a key role in recognizing PAMPs and subsequent immune signaling. Immature DCs are sentinel cells of the innate immune system that upon recognition and endocytosis of PAMPs mature and instruct the adaptive immune system, notably T cells, to undertake action. Upon antigen presentation, MHC-II and costimulatory molecules on DCs bind T cells. This feature combined with DC-secreted cytokines leads to differentiation and proliferation of T-cell subsets such as T helper (Th)-1, Th-2, and Th-17 cells (4, 14). The exact role that different mushroom compounds play in DC–T cell interactions remains unclear.

Edible mushrooms are non-invasive fungi that exert their immune effect in the absence of pathogenesis. Mushroom PAMPs may be recognized by DCs guarding the lumen of the gut, the lamina propria upon passing the mucosal epithelium or elsewhere in the body when digested compounds enter circulation. Regardless of the location where mushroom PAMPs are recognized, little is currently known about the *modus operandi* of mushroom immunomodulation, notably the effect on DCs and T cells. The aim of this study is to compare the immunomodulatory capacity of several mushroom species that have been reported to have an effect on the immune system. Thereto we compared the effect of seven edible mushroom species on *ex vivo* immune cells. We first assessed the composition of these mushroom species and their ability to stimulate DCs. Next, we studied endocytosis of mushroom compounds when presented to DCs and assessed the role of carbohydrate binding PRRs. Subsequently, we studied how mushroom compound challenged DC’s affect T cells. It is ultimately shown that mushrooms can aid in polarizing T-cell differentiation; however, physical interaction between DCs and T cells is not required. These new insights are useful for applying immunomodulatory mushrooms to promote health. This may be achieved by strengthening the immune system in general as well as in strategies to combat microbial infections for instance by using mushrooms as adjuvant.

## Materials and Methods

### Mushroom Extracts

All mushrooms were obtained from V.O.F. de Rijk-Stenssen (Rossum, The Netherlands). Mushrooms were harvested, directly transported to our laboratory, and frozen at −20°C. The frozen mushrooms were lyophilized and ground to a fine powder that was used as a complete homogenate (CH) in the experiments described. Part of the CH was alcohol precipitated to obtain a fraction enriched in polysaccharides according to Ng and Yap (15). Briefly, hot water extraction was performed for 20 min at 121°C in an autoclave on CH followed by overnight precipitation (4°C) with 70% ice-cold ethanol. The ethanol precipitate was removed by centrifugation, lyophilized, and subjected to a second hot water extraction. The second hot water extract was centrifuged and the supernatant was subjected to overnight precipitation with 50% ethanol at 4°C. The precipitate was collected by centrifugation (3500 × *g*, 30 min), lyophilized and ground to a fine powder, stored at −20°C, and used as an alcohol-precipitated polysaccharide fraction in the experiments described. For stimulations, CH and AP were dissolved in PBS in a concentration of 25 mg/ml.

### Cell Culture

Bone marrow was obtained from the femur and tibia of 6- to 12-week-old C57BL/6 mice. Bone marrow-derived dendritic cells (BMDCs) were differentiated for 10 days as previously described (16) using 10% spent medium from murine GM-CSF-transfected X63 cells (17). X63-GM-CSF cells were kindly provided by Dr. M. Lutz (University of Erlangen-Nuremberg, Germany) with approval of Dr. B. Stockinger (Francis Crick Institute, London, UK). Bone marrow cells were plated at 2 × 10^5^ cells/ml in bacteriological petri dishes (Greiner Bio-One, Alphen aan de Rijn, The Netherlands) and incubated at 37°C/5% CO_2_. At days 3, 6, and 8, medium was refreshed, and at day 10, both adherent and non-adherent cells were harvested. At this time, typically 90–95% of the cells expressed the DC markers CD11c and MHC class II as assessed by flow cytometry.

T cells were obtained from spleens of 6- to 12-week-old C57BL/6 mice. Isolation of the CD4+ T cells was done *via* the MagCellect Mouse CD4+ T Cell Isolation kit (R&D systems, Minneapolis, MN, USA) according to the manufacturer’s protocol.

Bone marrow-derived dendritic cells (5 × 10^5^ cells/ml) were stimulated in a 96-well plate (Greiner Bio-One) for 24 h upon addition CH or AP of different mushroom species to a final concentration of 250 μg/ml. Curdlan and zymosan were used as control stimulants in a final concentration of 50 μg/ml and 1 mg/ml, respectively. In CLR neutralization studies, 5 mM EGTA (Sigma-Aldrich, Zwijndrecht, The Netherlands) or 250 μg/ml laminarin (Sigma) was added 30 min prior to stimulation with the CH of different mushroom species. In a coculture of BMDCs (10^5^ cells/ml) and total T cells (5 × 10^5^ cells/ml) in 96-well plates, BMDCs were stimulated for 24 h where after T cells were added. Costimulation was done for 7 days.

The coculture in ThinCerts using 24-well plates (Greiner Bio-One) was done with 200 μg/ml CH, 250 μl DCs (2 × 10^5^ cells/ml) and 250 μl T cells (1 × 10^6^ cells/ml). During this experiment, there were two different setups. The control setup allowed the physical interaction between DCs and T cells, whereas the test setup did not. In both cases, a semipermeable membrane (ThinCert, 3 μm pore size, Greiner Bio-One), that allowed the exchange of medium and cytokines but not cells, was used. T cells were added 24 h after DC stimulation. In the test setup, the T cells were put into the “membrane cup” instead of in the well to avoid migration of DCs toward the T cells.

### Determination of Mushroom Composition

Mushroom α- and β-glucan composition was determined using a β-Glucan (yeast and mushroom) Assay Kit (Megazyme, Bray, Ireland) according to the manufacturer’s protocol. The amount of protein in the mushroom preparations was determined using a BCA protein assay (Life Technologies Europe BV, Bleiswijk, The Netherlands). Polyphenols were determined using a Folin Ciocalteu assay. Thereto, 30 μl Folin Ciocalteu’s phenol reagent (Sigma) was added to 15 μl sample. This was incubated for 7–8 min where after 75 μl of a solution of 20% anhydrous Na_2_CO_3_ was added. The amount of polyphenol was determined by measuring the absorbance at 550 nm using a standard line of gallic acid. Chitin content was determined as reported (18). In short, freeze dried mushrooms were heated using a water bath for 2 h in 1 M NaOH under continuous stirring. The sample was filtered and washed with distilled water. The procedure was repeated for 3 h using saturated KOH. The samples were centrifuged at 8000 × *g*, the pellet washed with distilled water, lyophilized and dissolved in 10% acetic acid, and heated at 100°C for 1 h. Of each sample, 2 μl was spotted on a thin layer plate (Polygram Sil G 0.2 mm, Macherey Nagel, Düren, Germany). The spots were detected with 1% Lugol’s solution, after color formation the plates were dried with hot air. The density was analyzed from an image using Image J. A dilution series of chitin (Sigma) was used as reference.

### Cytokine ELISA

TNF-α, Interleukin (IL)-6, IL-10, IL-12, IL-17, and IFN-γ levels in cell culture supernatants were determined using sandwich enzyme linked immunosorbent assay (ELISA) with ELISA Ready-Set-Go! Sets (eBioscience, Vienna, Austria) according to the supplier’s protocols.

### Flow Cytometry

Bone marrow-derived dendritic cells were stained in FACS buffer (PBS containing 0.1% bovine serum albumin and 5 mM EDTA) using the following monoclonal antibodies in the indicated concentration for cell surface markers (all obtained from eBioscience): PE-conjugated anti-CD86 (2 μg/ml), FITC conjugated anti-CD-40 (5 μg/ml), PE-conjugated anti-CD11c (1 μg/ml), and APC-conjugated MHC-II (0.28 μg/ml). Cells were first incubated with Fc receptor block, 5 μg/ml (eBioscience) for 10 min to block any non-specific binding and subsequent staining steps were performed for 20 min at 4°C, followed by washing with FACS buffer. An isotype-matched control staining was done for each antibody to determine a specific background staining. Cells were acquired using a Cyan-ADP cytometer (Beckman Coulter, Woerden, The Netherlands) and analyzed with FlowJo software (FlowJo LLC, Ashland, OR, USA).

### Analysis of Polysaccharide Endocytosis by Flow Cytometry and Confocal Microscopy

Polysaccharides from alcohol-precipitated preparations were labeled with Alexa Fluor 488 hydrazide (Invitrogen, Life Technologies) according to the manufacturer’s protocol. Binding of the labeled mushroom compounds to BMDCs was first assessed using flow cytometry using 5 × 10^5^ cells/ml and 5 μg/ml-labeled polysaccharides. Cells were acquired using a Cyan-ADP cytometer (Beckman Coulter). Next, endocytosis of polysaccharides was visualized by seeding BMDCs on borosilicate chambered coverglasses at a density of 5 × 10^4^ cells/chamber followed by incubation with Alexa Fluor 488-labeled stimulants (10 μg/ml-labeled polysaccharides of *Agaricus subrufescens* or *Lentinula edodes*). As a positive control, 10 μg/ml FITC-labeled dextran (Sigma) was used. Unstimulated cells were used as a negative control. After 6 h, the stimulants were removed by refreshing the medium. Confocal images were taken 24 h after the start of stimulation. Plasma membranes were stained with Cellmask Deep Red plasma membrane stain (Invitrogen, Life Technologies) and lysosomes with Lysotracker Red DND-99 (Invitrogen, Life Technologies). Imaging was done using an Axiovert LSM510 confocal microscope (Carl Zeiss, Sliedrecht, The Netherlands) using a water objective C-Apochromat 40×. Images were processed using the LSM Image browser (Carl Zeiss), release 4.2.

### Statistical Analysis

For statistical analysis, independent-samples *T* test was used. Analysis was carried out using SPSS version 22 (IBM corporation, Armonk, NY, USA).

## Results

### Composition of Mushroom Extracts

Before assessing immunomodulatory effects of CHs and alcohol precipitates (APs) of the seven mushroom species under investigation the amounts of protein, α- and β-glucan, chitin, and polyphenol were assessed as percentage dry weight (Table 1). The obtained results emphasize a great variety in composition between the different mushroom species. The chitin and polyphenol contents remained below 5% (w/w) for all preparations, whereby chitin was never detected in AP. Because chitin is insoluble and the procedure included a centrifugation step, this result was to be expected. The polyphenol contents increased upon ethanol precipitation for *A. subrufescens, L. edodes*, and *Hypsizygus tessellatus*. This result may indicate that these three species contain relatively high amounts of polyphenol/polysaccharide complexes or that the composition of the polyphenols differs. In the other four species, such complexes may still be present but to a much lower extent.

**Table 1:** **Mushroom composition**.

		Protein%	Total glucan%	α-glucan%	β-glucan%	Chitin%	Polyphenol%
*A. subrufescens*	CH	18.2	12.8	0.6	12.2	2.3	0.9
	AP	5.5	40.0	8.2	31.8	0	2.1
*G. frondosa*	CH	19.3	10.4	2.5	7.9	ND	4.4
	AP	24.5	38.5	10.3	28.2	ND	1.0
*L. edodes*	CH	5.8	21.4	1.5	19.9	1.97	2.3
	AP	0.0	34.2	7.0	27.2	0	4.3
*H. tessellatus*	CH	5.3	36.6	5.2	31.3	0.54	1.4
	AP	0.0	81.8	11.3	70.5	0	2.1
*P. eryngii*	CH	12.9	37.6	1.6	36.0	4.67	0.4
	AP	1.6	78.1	13.0	65.1	0	0.2
*P. nameko*	CH	9.0	59.5	1.4	58.1	1.32	3.3
	AP	11.7	36.8	0.9	35.9	0	0.3
*P. ostreatus*	CH	15.6	32.9	1.3	31.6	1.02	2.1
	AP	2.3	36.6	9.6	27.0	0	1.0

*The contents of protein, total glucan, α- and β-glucan, chitin, and polyphenol as percentage of the total dry weight are given. ND is not determined*.

The amounts of protein, α-, and β-glucan varied between preparations and between species. *Pholiota nameko* contained the highest amounts of β-glucan in the CH; however after ethanol precipitation, both the α- and β-glucan content was reduced. This is in contrast to what was expected as upon ethanol precipitation the glucan fraction is often enlarged which was the case for all other preparations. The most striking effect of the ethanol precipitation was seen for *H. tessellatus* and *Pleurotus eryngii* where the total amount of glucan increased from, respectively, 45 and 40% to around 80% of the dry weight. Upon ethanol precipitation, the protein content decreased in five of the seven species; only for *P. nameko* and *Grifola frondosa*, the protein content increased. This may be caused by a relative high amount of saccharopeptides present in these species. In the AP of *L. edodes* and *H. tessellatus*, no protein was detected. This result could indicate that in these two species no protein is bound to polysaccharides that are precipitated with ethanol. All in all, protein as well as α- and β-glucan contents vary greatly between species.

### Extracts from Different Mushrooms Species Lead to a Differential Cytokine Secretion Pattern in Dendritic Cells

**Figure 1:**
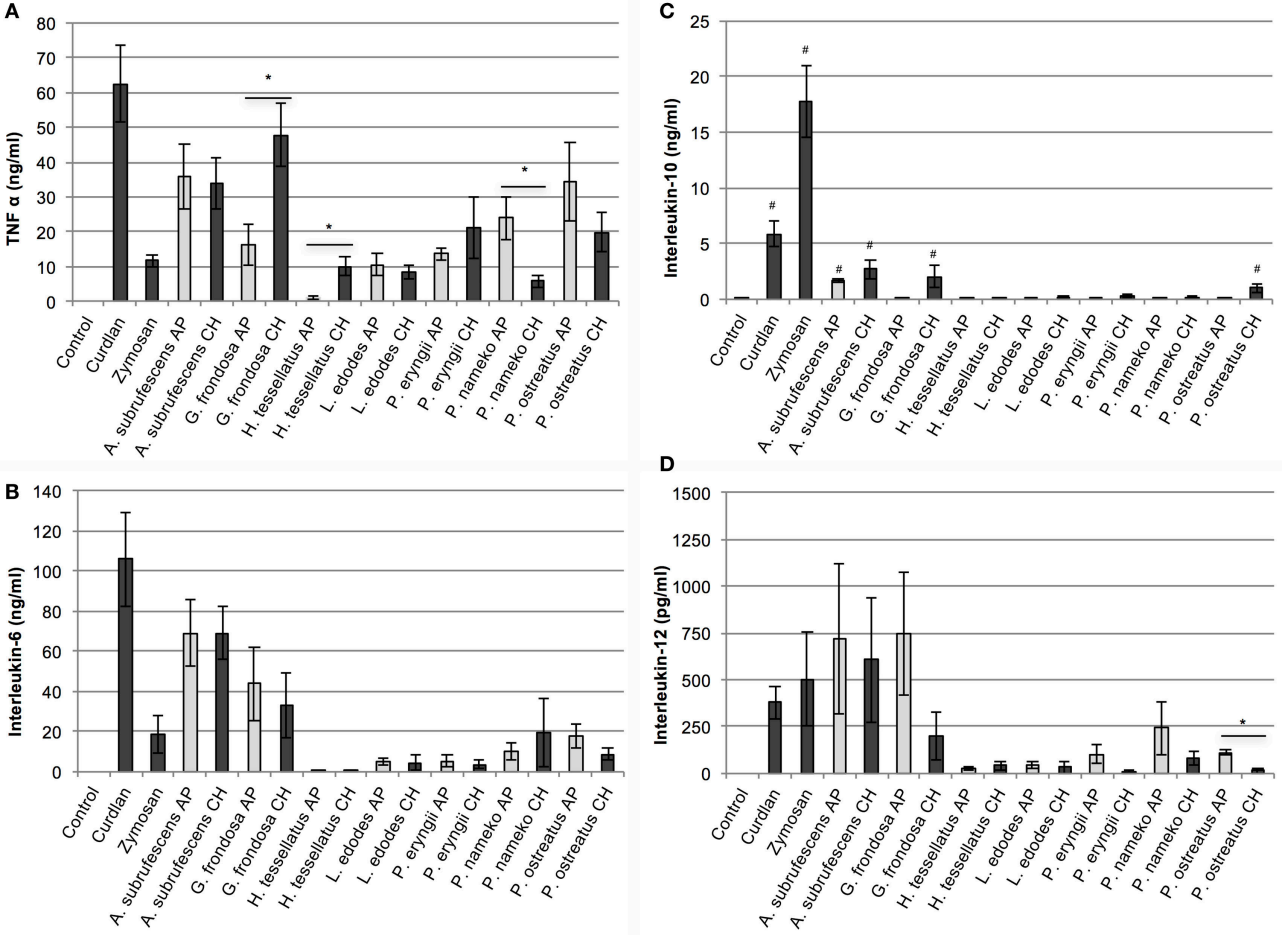
**The effect of mushroom compounds of seven mushroom species on cytokine secretion of bone marrow-derived dendritic cells**. Both the effects of the complete homogenate (CH) and the alcohol precipitate (AP) were analyzed. Curdlan and zymosan were used as controls. Both the mushroom species and the preparation methods show a differential effect on cytokine secretion by dendritic cells. **(A)** Tumor necrosis factor α. **(B)** Interleukin-6. **(C)** Interleukin-10. **(D)** Interleukin-12. Asterisk (*) indicates significant difference between CH and AP responses (*p* < 0.05, *n* = 4–7). Hashtag (#) indicates significant differences between control and mushroom compounds with regard to IL-10 secretion (*p* < 0.05, *n* = 4–7).

To determine the effect of mushroom compounds on cytokine expression by DCs, CH or AP of each of the seven mushroom species was added to DCs. ELISA was used to measure the effect on the secretion of cytokines TNF-α, IL-6, IL-10, and IL-12 (Figures 1A–D, respectively). In the control experiments, the expression of TNF-α, IL-6, and IL-12 remained below detection level. All preparations induced expression of the pro-inflammatory cytokines TNF-α and IL-6. *H. tessellatus* can be considered as the worst inducer of these cytokines. The CH of *H. tessellatus* induced TNF-α secretion in low amounts, which was almost absent upon stimulation with AP. *H. tessellatus* CH and AP hardly induced secretion of any of the other cytokines measured. It is worth noting that alcohol precipitation of *H. tessellatus* CH lead to the largest increase of glucan content, notably β-glucans (Table 1), whereas protein was absent in AP. Apparently, the *H. tessellatus* glucans cannot be considered as strong immunomodulators. Secretion of the anti-inflammatory cytokine IL-10 was significantly induced by CH of *A. subrufescens, G. frondosa*, and *Pleurotus ostreatus*. Of the AP preparations, only *A. subrufescens* significantly induced IL-10. Strikingly, the three species that induce IL-10 secretion show the highest protein content of all CH preparations (Table 1). Expression of the Th-1-inducing cytokine IL-12 was induced by *A. subrufescens* (CH and AP), *G. frondosa* (CH and AP), and *P. nameko* (AP). All other preparations induced IL-12 to concentrations <100 pg/ml. Considering all cytokines, alcohol precipitation, to enrich the polysaccharide fraction, affects the secretion of cytokines only to a minor extent. Only for *G. frondosa, H. tessellatus*, and *P. nameko*, the induction of TNF-α expression, and for *P. ostreatus*, the induction of IL-12 expression differed significantly between the CH and AP preparations (Figure 1). The seven species investigated have all been reported to immunomodulate. It was surprising to observe that *L. edodes* and *P. eryngii*, both species that have been reported to be strong immunomodulators, only moderately induced TNF-α and IL-6 secretion. In general, the extent to which the seven species investigated affect secretion of TNF-α, IL-6, IL-10, and IL-12 by DCs varies greatly and likely depends on the structure and composition of polysaccharide complexes either or not in combination with peptides/protein.

### Not All Mushroom Species are Potent Inducers of Maturation of Dendritic Cells

**Figure 2:**
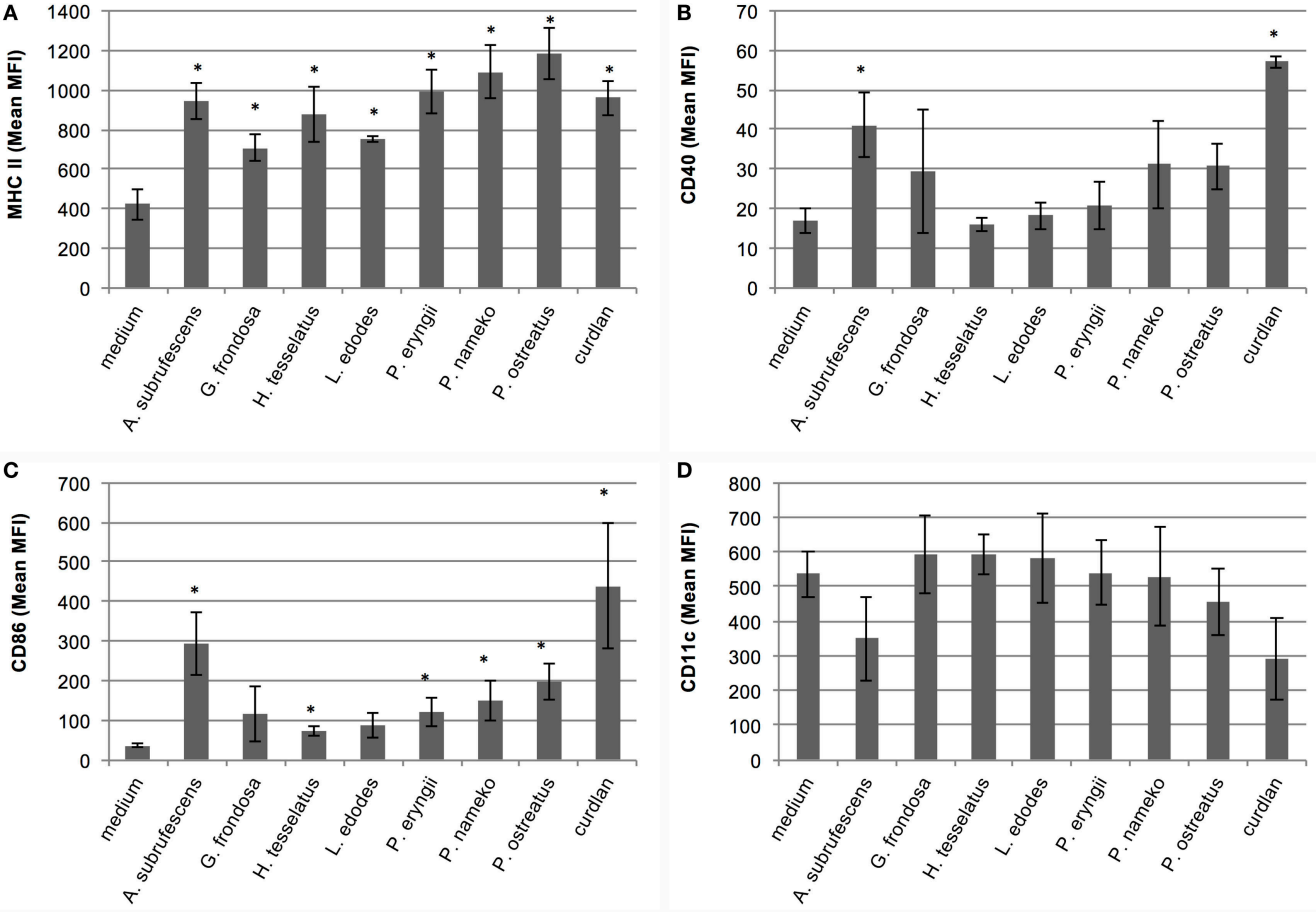
**Analysis of the maturation state of bone marrow-derived dendritic cells upon stimulation with compounds derived from seven mushroom species**. Dendritic cells (DCs) were stimulated with alcohol-precipitated mushroom compounds and curdlan as control. After 24 h, the DCs were labeled with fluorescently labeled antibodies and analyzed with flow cytometry. The obtained mean fluorescence intensity (MFI) is indicated. Only upon stimulation with *A. subrufescens*, mature DCs are observed. Notably, the upregulation of CD40 and CD86 and the downregulation of CD11c vary. **(A)** Labeling with anti-CD11c-PE. **(B)** Labeling with anti-CD40-FITC. **(C)** Labeling with anti-MHC-II-APC. **(D)** Labeling with anti-CD86-PE. Asterisk (*) indicates significant difference with the medium control, i.e., unstimulated DCs (*p* < 0.05, *n* = 3).

The differences in cytokine responses and notably the low responses induced by several species prompted us to study the maturation of the DCs. Thereto, DCs were stimulated with AP of each of the seven mushrooms. After 24 h the cells were labeled with anti-CD11c-PE, anti-MHC-II-APC, anti-CD40-FITC, or anti-CD86-PE antibodies and the fluorescence intensity was measured with flow cytometry. The average mean fluorescence of three independent experiments is depicted in Figure 2. Untreated cells, incubated in tissue culture medium, showed a typical immature BMDC phenotype, i.e., CD11c^+^-CD40^low^-CD86^low^-MHC-II^low^. Striking differences in the maturation state of the DCs stimulated with the AP from different mushrooms were observed. All mushrooms significantly upregulated MHC-II expression (Figure 2A). The expression of the costimulatory molecule CD40 was only significantly affected by *A. subrufescens* AP and curdlan (Figure 2B). In the case of the costimulatory molecule CD86, all mushrooms except *G. frondosa* lead to significant upregulation (Figure 2C). It was noted that the extent of CD86 upregulation differed considerably between species. Upon maturation CD11c is expected to be downregulated. However, considerable downregulation of CD11c was only observed for *A. subrufescens* AP and curdlan, although not significant (Figure 2D). Taken together, these data reveal that compounds from different mushrooms do affect DCs as revealed by the MHC-II and CD86 expression. However, CD11c and CD40 expression is much less affected. *A. subrufescens* is the most potent inducer of DC maturation.

### Dendritic Cells Bind and Endocytose Mushroom Compounds

**Figure 3:**
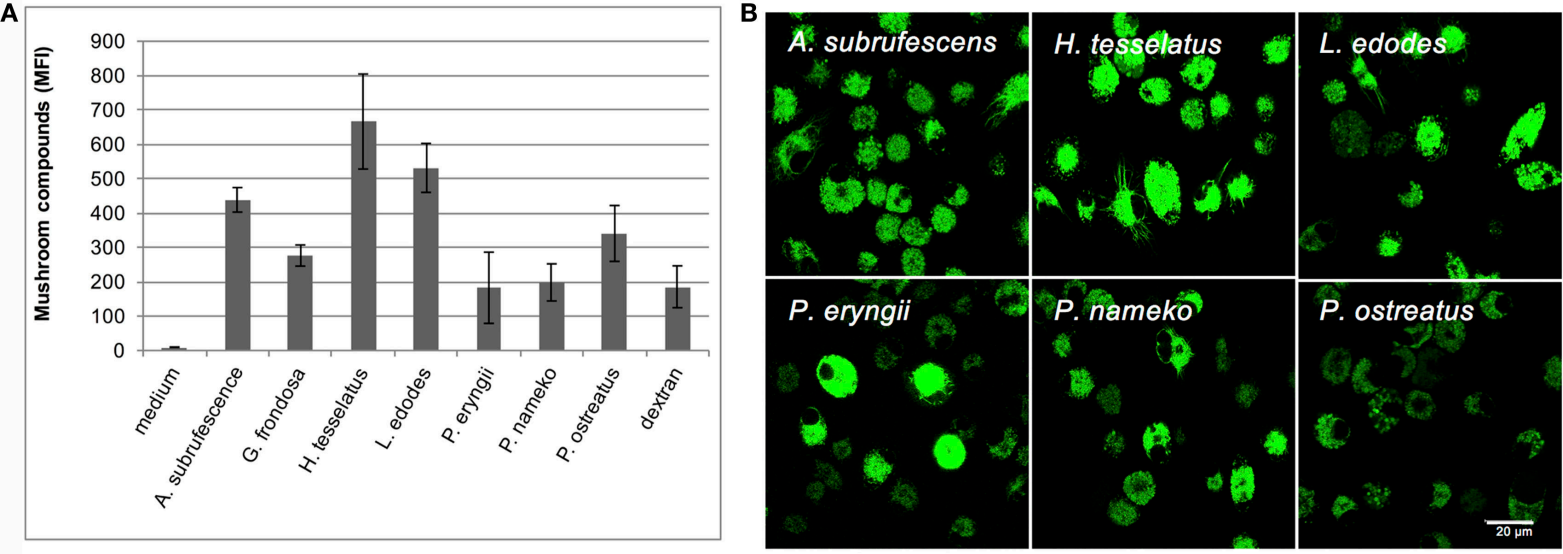

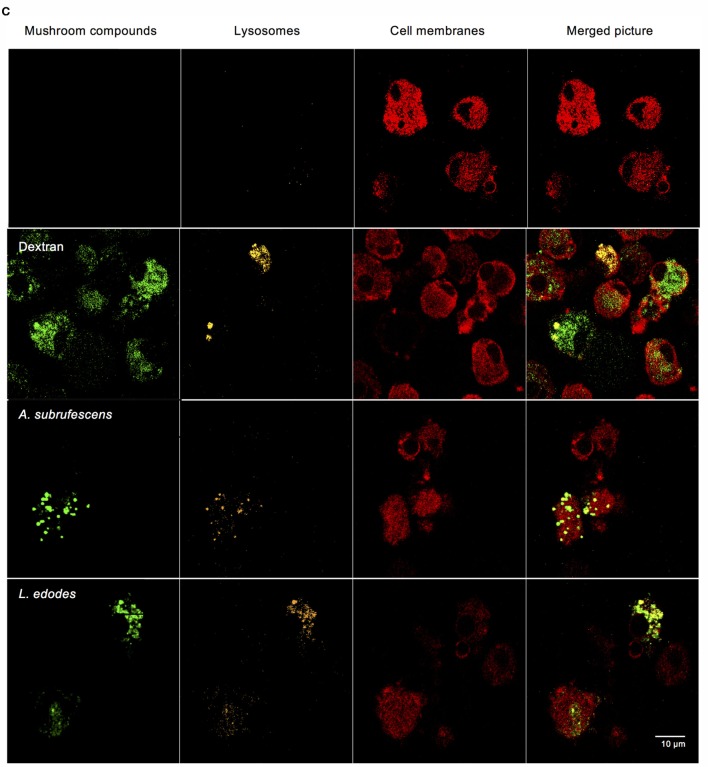
**Dendritic cells endocytose mushroom compounds**. Alexa Fluor 488-labeled mushroom compounds were added to DCs and analyzed by flow cytometry and confocal microscopy. Fluorescent dextran was used as control. **(A)** Binding of fluorescently labeled mushroom compounds to DCs as analyzed by flow cytometry. **(B)** Endocytosis of fluorescent mushroom compounds of six mushroom species analyzed with confocal microscopy. **(C)** Endocytosis of fluorescent *Agaricus subrufescens* and *Lentinula edodes* compounds visualized using confocal microscopy (40× magnification). Plasma membranes and lysosomes were stained as indicated. The merged pictures reveal that labeled compounds are present in the endo-lysosomes of DCs.

Because of the variation in the extent of cytokine secretion and maturation, and notably the weak responses observed for *H. tessellatus, L. edodes*, and *P. eryngii*, we investigated whether or not mushroom compounds are recognized by DCs in all cases. To investigate this, Alexa Fluor 488-labeled mushroom compounds and FITC-labeled dextran as control were added to DCs. Because Alexa Fluor 488 hydrazide was used, it is expected that only polysaccharides and glycoproteins were labeled. Using flow cytometry we confirmed that labeled compounds from all mushrooms investigated as well as dextran bind DCs; however, the amounts vary considerably (Figure 3A). Using confocal microscopy, we confirmed that compounds of all mushrooms investigated are indeed endocytosed (Figure 3B). We next investigated endocytosis by DCs in more detail using *A. subrufescens* as the best responder and *L. edodes* as weak responder. The labeled mushroom compounds showed indeed to ultimately reside in the (endo-)lysosomes (Figure 3C). Both protein and glucan composition vary considerably as do the amounts that bind DCs (Figure 3A). Nevertheless, endocytosis was observed in all cases (Figure 3B). We therefore next investigated the role of CLRs as carbohydrate binding PRRs.

### PRRs Specific for Carbohydrates Bind Mushroom Compounds

**Figure 4:**
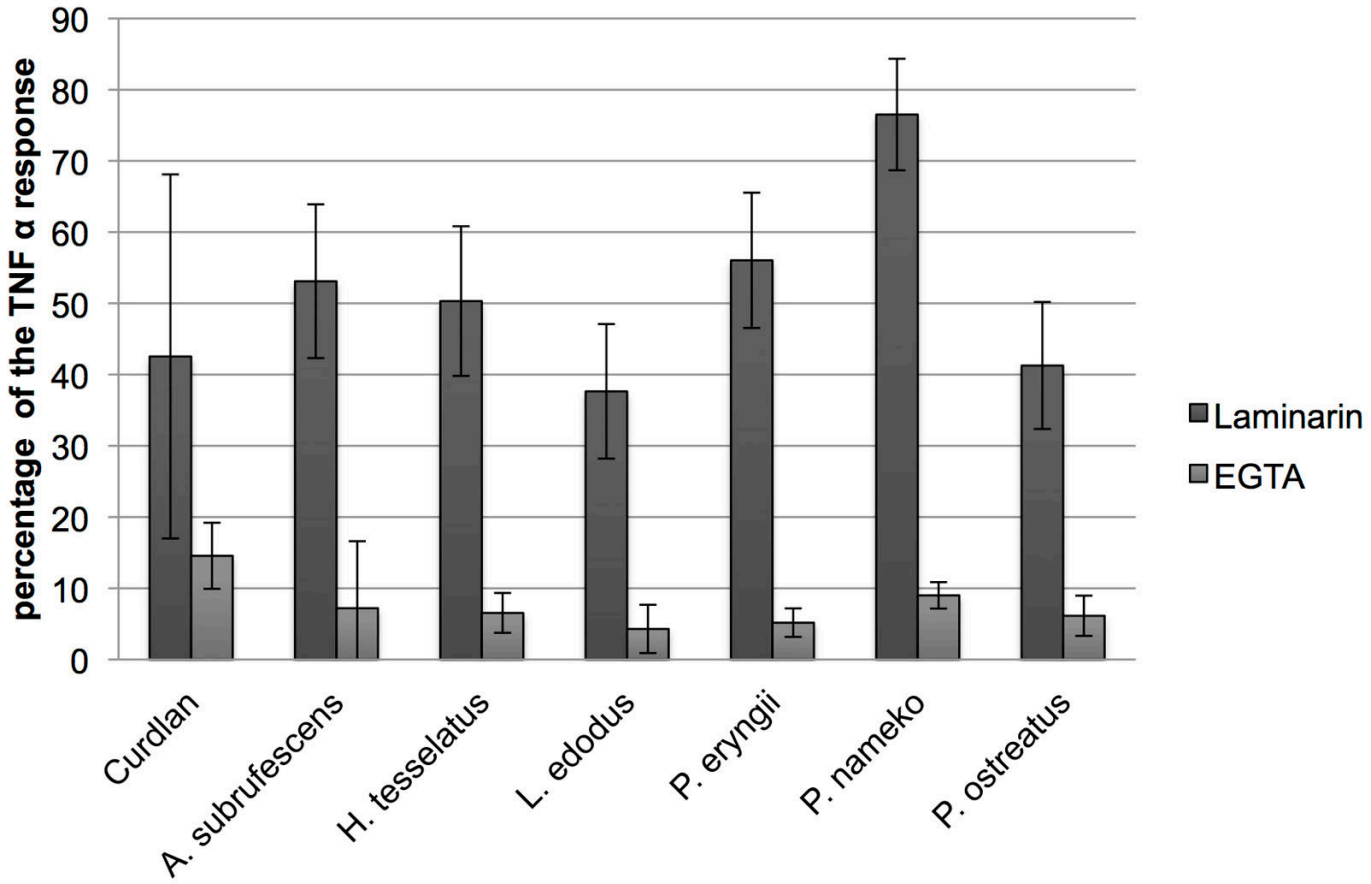
**C-type lectin receptors and notably dectin-1 bind mushroom compounds**. Dendritic cells were pre-incubated with either the chelator EGTA or the low molecular weight soluble β-glucan laminarin followed by stimulation with mushroom compounds. The effect of EGTA and laminarin was assessed by measuring the effect on the TNF-α response. EGTA reduced the TNF-α response to 5–10% of the original response and laminarin showed an inhibition to 40–75%. The effect of EGTA indicates that this class of PRRs plays an important role in DC stimulation because C-type lectin receptors (CLRs) are calcium dependent. Laminarin is a dectin-1 antagonist and shows that this CLR is involved in the binding of mushroom compounds.

Because mushrooms contain a substantial amount of carbohydrates that may function as PAMP the involvement of carbohydrate-specific PRRs in endocytosis seems plausible. CLRs and CR3 are major endocytosis receptors as well as an important carbohydrate binding class of PRRs that function in a calcium-dependent manner. The addition of a chelating agent inhibits carbohydrate binding to CLRs and CR3. To investigate CLR/CR3 involvement, DCs were therefore pre-incubated with the chelating agent EGTA, whereupon CH of six mushroom species (*A. subrufescens, H. tessellatus, L. edodes, P. eryngii, P. nameko*, and *P. ostreatus*) was added. After 24 h, the TNF-α response was measured. EGTA inhibits the TNF-α response with 90–95% (Figure 4). The inhibition of the cytokine response by EGTA suggests that mushroom compounds do indeed target CLRs and/or CR3.

β-Glucans are polymeric carbohydrates assumed to play an important role in immune stimulation by mushrooms through the CLR dectin-1 (2, 6). Laminarin is a low molecular weight water-soluble β-glucan that binds dectin-1 without stimulating downstream signaling, yet blocking binding of other β-glucans to dectin-1 (19). Pre-incubation of DCs with laminarin inhibits the TNF-α response with 25–60% (Figure 4). Therefore, we conclude that dectin-1 binding of β-glucans plays an important role in the stimulation of DCs by mushrooms. Dectin-1 is, however, not the only carbohydrate binding PRR involved as can be inferred from the differences in inhibition by laminarin.

### Mushrooms Stimulate a Mixed CD4+ T-Cell Response

**Figure 5:**
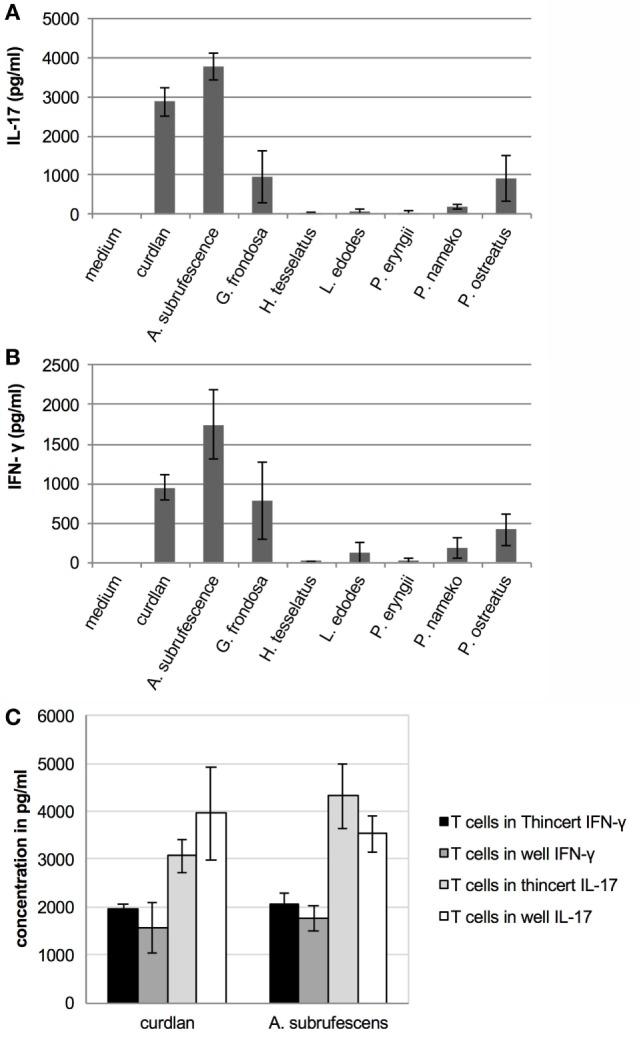
**Mushrooms stimulate T cells through DCs; however, physical contact between these cells is not required**. T cells were added to CH stimulated dendritic cells that were physically separated from each other or not. Seven days later the interferon-γ (IFN-γ) and interleukin-17 (IL-17) response was measured. **(A)** Mushroom induced IL-17 response. **(B)** Mushroom induced IFN-γ response. **(C)** Regardless of whether or not T cells were physically separated from DCs (when placed in a ThinCert), the IFN-γ and IL-17 responses do not significantly differ.

Since mushroom compounds are endocytosed, induce cytokine secretion, MHC-II expression and in some cases expression of CD40 and CD86 molecules, we next assessed the effect of mushroom-stimulated DCs on T cells. Thereto, T cells were added to DCs that were stimulated for 24 h with CH of different mushrooms. After 7 days, the presence of the T-cell cytokines IFN-γ, IL-4, and IL-17 was assessed using ELISA. The incubation of T cells with mushroom extracts for 7 days never resulted in the secretion of IFN-γ, IL-4, and IL-17 (data not shown). In the cocultures, detectable levels of IL-4 were never measured (data not shown). However, secretion of IL-17 and IFN-γ (Figure 5) was stimulated by *A. subrufescens, G. frondosa, L. edodes, P. nameko*, and *P. ostreatus*, although secretion of IL-17 induced by *L. edodes* was low. IFN-γ and IL-17 are the signature cytokines for a Th-1 and Th-17 response, respectively. These results suggest that mushrooms are capable of stimulating a mixed T-cell response, as seen for many pathogens. As mushrooms are (non-pathogenic) fungi, it was expected that a Th-17 response would prevail alike what has been shown for pathogenic fungi.

### Physical Contact between DCs and T Cells Is Not Required to Stimulate T Cells

Although mushroom compounds can stimulate T cells through DCs, the question that remains is whether or not DCs process endocytosed mushroom compounds and present fragments to T cells *via* MHC-II molecules. To date, only the zwitterionic polysaccharide A of *Bacteroides fragilis* has been shown to activate T cells in a MHC-II dependent manner (20). Other polysaccharides stimulate the immune system in a T cell-independent manner. Since the preparations of mushroom compounds also contain protein, these may be presented to T cells *via* MHC-II molecules. If presentation through MHC-II molecules takes place, physical contact between DCs and T cells is required. An experiment was carried out wherein DCs and T cells were physically separated by using ThinCert™ membrane supports. DCs were stimulated with *A. subrufescens* CH for 24 h in a 24-well plate, when a ThinCert™ membrane support holding T cells was placed in the well. Simultaneously, DCs were incubated together with T cells in a 24-well plate. *A. subrufescens* was chosen for this experiment as this mushroom showed the strongest effect on DCs and T cells with regard to cytokine secretion as well as on the upregulation of the costimulatory molecules CD40 and CD86. After 7 days, the secretion of IFN-γ and IL-17 was determined using ELISA. Physical contact between DCs and T cells was not necessary to stimulate T cells to secrete IFN-γ and IL-17 (Figure 5C). Hence, fragments of mushroom proteins likely do not mediate the stimulation of T cells as this would be mediated by MHC-II molecules. This result implies that (polymeric) mushroom carbohydrates activate DCs resulting in cytokine secretion that stimulate T cells.

## Discussion

In this study, we evaluated the immunomodulatory effect of seven edible mushroom species on *ex vivo* immune cells. We used CHs, resembling mushrooms as part of the human diet, as well as APs of the CH. Alcohol precipitation generally increases the polymeric carbohydrate fraction, including glucans that have been identified as important immunomodulators (4). The secretion of cytokines by DCs and CD4+ T cells was assessed as well as DC maturation and endocytosis of mushroom compounds by DCs. We demonstrated that the CH and AP of different mushroom species had varying effects on *ex vivo* cells, and that carbohydrate receptors (CLRs and CR3), notably dectin-1, are important PRRs in mushroom immune stimulation. Physical contact between DCs and T cells was not required to stimulate CD4+ T cells.

Variation in DC responses may be explained by differences in mushroom composition as well as by different compounds that, either or not in concert, affect immune cells. We determined the α- and β-glucan-, protein-, chitin-, and polyphenol-content as these compounds are known to have immunomodulatory effects. Nevertheless, the IL-6 and IL-10 responses of both CH and AP preparations were similar for all species. The fact that EGTA almost completely abolishes the TNF-α response indicates that carbohydrates play a dominant role in mushroom immunomodulation. The TNF-α response of *G. frondosa* and *H. tessellatus* differed significantly, with the CH preparations resulting in a stronger response than the AP preparations. In contrast, the TNF-α response of the AP of *P. nameko* and the IL-12 response of the AP of *P. ostreatus* were stronger than their respective CH preparations. *P. nameko* was the only mushroom for which alcohol precipitation did not increase the glucan content. Thus, for this species, the increase in polysaccharide content does not coincide with an increase in cytokine response.

Immunomodulating carbohydrate containing compounds found in mushrooms vary in structure and composition. Mushroom β-glucans are structurally diverse molecules varying in branching, through 1,6 linkages, and in molecular mass (21, 22). β-Glucans with a degree of branching between 0.20 and 0.33 seem to be the most active (23). It may well be that in the low-responding mushrooms investigated, the fraction of active β-glucans is low. It has been reported that many biotic and abiotic factors affect the synthesis and/or degradation and consequently the structure of β-glucans. In *L. edodes*, increased glucanase activity during storage affected the cytokine response of macrophages (24, 25). Genetic factors may also play a role as was shown for different strains of *L. edodes* that showed a different effect on inhibition of NO production by macrophages (26). The *L. edodes* mushrooms, highly appreciated for their immunomodulating properties, induced relatively low cytokine responses (Figure 1). This may be explained by variations in structure and composition of polysaccharides. Proteoglycans and glycoproteins are other molecules that contribute to immunomodulation by mushrooms. For *A. subrufescens* (also known as *Agaricus blazei*), water-soluble proteoglycan was reported to stimulate BMDCs (27). Also in our hands, *A. subrufescens* showed to be a strong immunomodulator that may well be caused by carbohydrate containing proteins. Taken together, the variation in mushroom compound structure and composition largely determines the immunomodulatory activities resulting in a distinct stimulation of immune cells.

Mushroom stimulation of DCs leads not only to distinct cytokine secretion patterns but also to distinct maturation states of DCs (Figure 2). The cytokine secretion patterns match the maturation state of DCs. Mushroom species that elicit poor cytokine responses also lead to reduced upregulation (MHC-II, CD40, CD86) or downregulation (CD11c) of maturation markers. It is well established that upon immune stimulation, a spectrum of maturation states of DCs is seen, varying from immature to semi-mature and mature (28). The maturation state is rather a phenotypic description than a functional description and does not necessarily coincide with the activation state of a DC (29). DCs that are phenotypically mature can still lead to highly varying T-cell responses. The activation state of a DC is determined by the combination of expressed membrane receptors and cytokines that lead to activation of T cells. *In vivo* the activation state of DCs is instigated by a combination of signals. These are signals received from PAMPs that bind PRRs, autocrine and paracrine effects of PRR induced cytokines, feedback signals from other innate, and adaptive immune cells as well as danger signals (30). Direct recognition of PAMPs by DCs is the crucial first step that is required to elicit proper T-cell responses. The other signals are merely amplification signals to ascertain that an immune response is of an appropriate magnitude (31). In the *in vitro* setup used in this study, *ex vivo* BMDCs are only stimulated by mushroom PAMPs possibly combined with some autocrine cytokine stimulation. Paracrine signals are excluded, and the contribution of direct PRR signaling could be assessed. Hence, the differences in activation state of the *ex vivo* DCs can only be caused by differential binding of mushroom PAMPs to PRRs.

Polymeric carbohydrates, glycoproteins, and proteoglycans are important carbohydrate containing fungal immunomodulators that mediate their effect by binding to different classes of PRRs (32). Most, if not all, of these compounds are important building blocks of the fungal cell wall and contain, next to glucose, mannose and, to a minor extent, other carbohydrates such as galactofuran. By using EGTA, it was revealed that preparations of all mushroom compounds bind carbohydrate binding PRRs, i.e., CLRs and CR3 (Figure 4). As recently published, particulate β-glucans are required to activate dectin-1 (33). We showed that the low molecular weight and soluble β-glucan laminarin inhibits immunomodulation by mushroom compounds. This observation implies that the β-glucans in the mushroom preparations are of a particulate nature. Since EGTA and laminarin never completely reduced TNF-α responses, other than β-glucan mediated responses have to be considered as well. Mannose-containing compounds are also expected to be present in the preparations that we used, and they also bind CLRs such as the mannose receptor, DC-SIGN, and galectins. Mannose is present in the glycans attached to proteins as well as in mannan, a fungal polysaccharide. Mannan containing compounds also bind TLR2 and TLR4, and likely TLR2/4 and TLR2/6 heterodimers (34). These TLRs have been reported to play a role in anti-fungal immunity (34). *In vivo*, the engagement of a ligand by a TLR is a prerequisite for DC maturation (35). Mushrooms can induce activation of DCs through TLRs as was shown for *Sparassis crispa* and *Phellinus linteus*. For *S. crispa*, the involvement of TLR4 was shown (15), whereas for *P. linteus*, the involvement of TLR2 and TLR4 was proven (36, 37). *A. subrufescens* was shown to bind TLR2 on THP-1 cells, and in the same study, the same preparation induced maturation of human monocyte derived DCs (38). Hence, a role for TLR2 in the activation of DCs by *A. subrufescens* seems plausible. The complex nature of whole-mushroom extracts, combined with the fact that different PRRs play a prominent role, may well lead to additive or even synergistic effects as pointed out previously (13). In studies of the immune response against pathogenic fungi, synergistic effects have indeed been reported (34, 39).

The fact that MHC-II expression is upregulated by mushroom compounds raised the question what the effect of the mushroom-stimulated DCs on CD4+ T cells is. In the DC–T-cell coculture experiments, five of the seven mushroom species stimulated the secretion of varying amounts of T-cell cytokines (Figure 5). Yet, we never observed any visible proliferation of T cells despite the fact that around 70% of the splenic CD4+ T cells were naïve (data not shown). Physical separation of DCs and T cells did not affect the results with regard to the secretion of IFN-γ and IL-17 (Figure 5C). Apparently, as non-pathogenic microorganisms, mushrooms do not lead to antigen presentation even when DC activation is induced, as was the case for *A. subrufescens*. It is therefore somewhat puzzling that five of the seven mushrooms lead to the production of IFN-γ and IL-17. Bystander activation of resting T cells has been reported (40). In the presence of IL-2, TNF-α, and IL-6 resting T cells do show some proliferation, albeit still poor with only 3% of the cells growing. The amounts of IFN-γ and IL-17 produced by the T cells are congruent with the amounts of TNF-α and IL-6 produced by the DCs upon stimulation by the seven mushroom species. The minor proportion of splenic T cells that were already activated may well have produced the required IL-2 for the small, but imperceptible, number of T cells that proliferated in the coculture. This mechanism of antigen-independent stimulation may explain why no contact between DCs and T cells is required. Even partially activated DCs, as seen for *G. frondosa, L. edodes, P. nameko*, and *P. ostreatus*, may contribute to a low level of bystander activation of T cells.

Taken together, we show that mushrooms can stimulate cells of the adaptive immune system without antigen presentation. Thus, mushrooms do not directly lead to T-cell activation through recognition of MHC-bound peptide by the antigen-specific T-cell receptor, often referred to as signal 1. However, not only this signal 1 is required for the activation of naïve T cells. Co-stimulation (signal 2) through other membrane receptors as well as cytokines and growth factors that direct Th cell stimulation (signal 3) are also required. Our results show that mushrooms, depending on the species, can stimulate signals 2 and 3 through their binding to CLRs possibly in combination with TLRs. Thus, mushrooms that upregulate signal 2 and 3 may well function as an adjuvant. This mechanism may explain how mushrooms exert their immunomodulating effects. A vast amount of literature describes immunomodulating effects of mushrooms; however, the mechanisms that result in immunomodulation remain obscure. Our findings disclose such a mechanism and may explain how mushroom consumption affects the immune system. In South and East Asia, mushrooms are often consumed upon cancer treatment by chemotherapy and/or radiotherapy. Both cancer and its treatments are immunosuppressive; hence, immunostimulating agents may be useful as long as they do not interfere with the conventional therapy (41). Another possible advantage of the regular consumption of mushrooms may be the reduction or alleviation of food allergies. Oral administration of a protein isolated from the edible mushroom *Flammulina velutipes* inhibited the development of an allergic reaction to the model food (egg) allergen ovalbumin in mice (42). A similar result was obtained with an extract from *A. subrufescens* (also known as *A. blazei*) and ovalbumin allergy (43). The same mushroom also protected mice against pneumonia caused by *Streptococcus pneumonia* upon oral administration of a water extract. The examples show that as non-invasive fungi, mushrooms are recognized by the mucosal immune system and affect the innate and adaptive immune system in a system-wide manner.

Currently there is a need for new adjuvants that are safe and efficient. Adjuvants targeting CLRs as an alternative to or in concert with TLRs may be beneficial for vaccine efficacy (44, 45). Adjuvants based on carbohydrates are particularly useful in the development of anti-fungal vaccines. Curdlan, a 1,3-β-glucan, showed to be a useful adjuvant when combined with a novel *Candida albicans* epitope. This β-glucan polarized the immune response in the direction of a Th-17 response (46). In the defense against other fungi, a combined targeting of TLRs and CLRs is required as was shown for *Fonsecaea pedrosoi* causing chromoblastomycosis, a chronic skin infection (47). Next to the general immune stimulating properties ascribed to mushrooms, these examples show that mushrooms may be useful in treating specific diseases. Thereto, mushrooms or mushroom preparations may be taken orally, although parenteral applications are also possible for these applications. Future, *in vivo* studies should show whether edible mushrooms could fulfill the promise as described.

## Ethics Statement

All animal experiments were conducted in compliance with the Dutch law and approved by the Experimental Animal Committee of Wageningen University.

## Author Contributions

RW contributed to the experimental design, data interpretation and the preparation of the manuscript. LW contributed to the data interpretation and preparation of the manuscript. JV designed and carried out the confocal microscopy experiment and interpreted the data of these experiments. GS contributed to the supervision, data interpretation and contributed to content of the manuscript. DR was responsible for the cell culture and ELISA experiments and determined the composition of the mushroom preparations. AsmS conceived the project, provided the mushroom preparations, reviewed part of the data that ensued from the cell culture experiments and reviewed the manuscript. JB contributed to the supervision and wrote parts of the manuscript. AS supervised and conceived the project and wrote the manuscript.

## Conflict of Interest Statement

The authors declare that the research was conducted in the absence of any commercial or financial relationships that could be construed as a potential conflict of interest.
